#  Psychometric Properties of the Persian Version of Cerebral Palsy Quality of Life Questionnaire for Children 

**Published:** 2015

**Authors:** Farin SOLEIMANI, Roshanak VAMEGHI, Anoshirvan KAZEMNEJAD, Nazila AKBAR FAHIMI, Zahra NOBAKHT, Mehdi RASSAFIANI

**Affiliations:** 1 Research Center, University of Social Welfare and Rehabilitation Sciences, Tehran, Iran; 2 Biostatistician, Tarbiat Modarress University, Tehran, Iran; 3 Occupational Therapy student, Department of Occupational Therapy, University of Social Welfare & Rehabilitation Sciences, Tehran, Iran

**Keywords:** Quality of life, Cerebral palsy, CP QOL-Child

## Abstract

**Objective:**

Cerebral palsy (CP) is the most common cause of chronic disability that restricts participation in daily life for children. Thereby, it is comprised of quality of life. Quality of life (QOL) measures have been a vital part of health outcome appraisals for individuals with CP and to obtain empirical evidence for the effectiveness of a range of interventions. The CP QOL-Child is a condition-specific QOL questionnaire designed for children with CP to assess well-being rather than ill-being.

**Materials & Methods:**

Forward and backward translations of the CP QOL-Child were performed for: (1) the primary caregiver form (for parents of children with CP aged 4–12 years); and (2) the child self-report form (for children with cerebral palsy aged 9–12 years). Psychometric properties assessment included reliability, internal consistency, and item discrimination, construct validity with Gross Motor Function Classification System (GMFCS) and Manual Ability Classification System (MACS) was done. SPSS was used to analyze the results of this study.

**Results:**

A sample of 200 primary caregivers forchildren with CP (mean = 7.7 years) and 40 children (mean = 10.2 years) completed. Internal consistency ranged from 0.61–0.87 for the primary caregivers form, and 0.64–0.86 for the child self-report form. Reliability ranged from 0.47–0.84. Item discrimination analysis revealed that a majority of the items (80%) have high discriminating power. Confirmatory factor analysis demonstrated a distinguishable domain structure as in the original English version. Moderate associations were found between lower QOL and more severe motor disability(GMFCS; r = .18–.32; p < .05 and MACS; r= .13 - .40; p < .05). The highest correlation between the primary caregiver and child forms on QOL was in the domain of functioning and consistent with the English version.

**Conclusion:**

Content validity, item discriminant validity, internal consistency, and test-retest reliability of the Persian version of the CP QOL- Child were all acceptable. Further study of concurrent validity of this version is needed.

## Introduction

Cerebral palsy (CP) describes a group of disorders in the development of movement and posture, causing activity limitation that is attributed to non-progressive disturbances that occur in the developing fetal or infant brain (1). It is the most common cause of chronic disability that restricts the participation in daily life for children (2-3), and thereby compromises quality of life (QOL) of children with CP (4-8). When compared to the typical development, children with CP have poorer QOL (4-8). The United Cerebral Palsy Association (1991) adopted the mission statement: ‘‘to affect positively the quality of life of persons with cerebral palsy.’’ QOL has become the most important outcome for treatments for children with CP (9). QOL measures have been a vital part of health outcome appraisals for individuals with CP to obtain empirical evidence for the effectiveness of a range of interventions. 

The CP QOL-Child is a condition-specific QOL questionnaire designed for children with CP to assess well-being rather than ill-being. It can be used to evaluate the effectiveness of treatment interventions on improving well-being (10). It has two parallel forms, a primary caregiver-proxy report for children aged 4-12 years of age and a self-report form for children aged 9-12 years of age. Three features are notable about the design of the CP QOL-Child as follows: (1) it is based on the International Classification of Function (ICF); (2) it has been developed with international expertise; and (3) it recognizes the importance of obtaining the views of the child and primary caregivers in developing and completing the questionnaire. The starting age of four years was chosen to ensure that the child was old enough to have a clear diagnosis of CP. Children aged over 12 years were not included as it is possible that new issues, such as body image, school pressures, and employment will emerge during adolescence. 

The measurement structure of the CP QOL-Child proxy version was examined in two previous studies (11- 12). In Iran, many researchers have conducted studies on outcome variables to evaluate the effectiveness of interventions for children with CP, but a measure of QOL for children with CP remains unavailable. As the CP QOL-Child is a well-developed measurement scale, it is meaningful to validate the Persian version. The aims of this study are to (1) translate the CP QOL-Child questionnaire into Persian; (2) assess the reliability of this translated version of CP QOL-Child; and (3) validate its use for children with CP in Persian- speaking communities. 

## Materials & Methods

A convenience sampling of children with CP and their primary caregivers was used to recruit the participants from rehabilitation departments and special education schools in Tehran from 2011–2012. Ascertainment of cases was based on a standard definition of CP (13). The inclusion criteria were as follows: Children aged between 4–12 years, and diagnosed with CP by a pediatric neurologist; capability of providing self-report or could understand the explanations of the questionnaire content; and their primary caregiver was capable of completing the questionnaires without any assistance. Children suffering from neurodegenerative diseases or psychiatric illness were excluded. 

Demographic data collected on primary caregivers included age, gender, educational level, marital status, and employment status. Data collected on children included age, gender, and intensity of rehabilitation intervention (times per week). Ethics approval was gained through the University Ethical Committee. Informed consent was obtained prior to data collection. 

**Measures**

For the first administration, primary caregivers (n=200) were given the CP QOL-Child and other questionnaires of functioning, as well as additional questions regarding demographics. The child self-report form was for children with CP aged 9–12 years (n = 40). A total of 40 children aged 9-12 years completed questionnaire, because only a proportion of children were able to complete the questionnaire due to age or severity of impairment. For the second administration, 20 families were randomly allocated to complete the CP QOL-Child for a second time, three weeks after the first administration.The instrument measures seven domains of QOL for a child with CP as follows: (1) social well-being and acceptance; (2) functioning; (3) participation and physical health; (4) emotional well-being; and (5) pain and impact of disability. The other two test domains were only included on the primary caregiver form as follows: (6) access to services; and (7) family health. 

The stem of the test item is “How do you think your child feels about...?” or “How do you feel about...?” This type of item stem was used because it does not measure the condition or functioning of the children, rather it assesses how they feel about their condition. The CP QOL-Child uses a Likert scale to measure happiness. All the items except one are rated on a nine-point scale ranging from 1–9. One item from the domain of pain and impact of disability is rated on a five-point scale. The primary caregiver-proxy form (children aged 4–12 y) contains 66 items and the child self-report form (children aged 9–12 y) contains 52 items (completion time for both ranged from 24–42 min). 

The CP QOL-Child is designed to provide several domain scores and all items are aggregated and averaged. All QOL scores were converted to a scale from 0–100. It was anticipated that scores on the CP QOL-Child would be moderately positively correlated with other measures of functioning and contributing evidence to its validity. The relationship between primary caregiver-proxy, and child self-report scores was examined as only a proportion of children were able to complete the questionnaire due to age or severity of impairment. 

The neuromotor type, topographic pattern, and severity of motor disability of the children with CP were assessed by direct observations of a clinician using the classification system developed by the Surveillance of Cerebral Palsy in Europe Collaborative Group (SCPE) (14), manual ability classification system (MACS), and the Gross Motor Function Classification System (GMFCS) (15- 16), respectively. Motor types were classified as spastic, dyskinetic, mixed, ataxic, and hypotonic (17-19). The GMFCS level, which varies from 1-5, is a measure of functional mobility and focuses on lower limb function (20, 15-16). It is based on self-initiated movement with particular emphasis on sitting and walking. Distinctions between the five levels of motor function are made on functional limitations and the need for assistive devices. Thus, children classified as level I have the most independent motor function, while children at level V have the least (21). 

MACS provides a systematic basis to classify how children with CP use their hands when handling objects were 

for daily activities. MACS is based on self-initiated manual ability, with particular emphasis on handling objects in an individual’s personal space. As a general principle, if the manual ability of a child fits within a particular level, then the child will probably be classified either at or above that level. Level I includes children with CP with, at most, minor limitations compared to typical development children, and where the limitations, if any, barely influence performance of daily life tasks. Distinctions between each pair of levels are also provided to assist in determining the level that most closely resembles the manual abilities of a child. The scale is ordinal, with no intent that the distances between levels should be considered equal, or that children with CP are equally distributed across all five levels (22-23). 

**Procedure**

Formal permission to translate the English version into Persian was obtained from the original authors. Then, the following steps were conducted to validate the tool in Persian according to the International Quality of Life Assessment (IQOLA) Project (24) as follows: (1) translation of each item into Persian by two native Persian-speaking people independently, who have knowledge of both English and Persian language and familiarity with the cultures and have experience in test development and CP; (2) reconciliation of items on two independent forward versions; (3) piloting of the translated items by inviting three parents of the children and three child with CP to fill the preliminary Persian CP QOL-Child; (4) a panel discussion to revise the problematic items; (5) examination of the revised Persian items; (6) backward-translation of the items into English; (7) review of any major differences in content between the backward-translated English items and the original English items; (8) mail the final English version to the original authors; additionally, the authors worked with the original developers of the questionnaire to ensure that the intended meaning was captured with the translation; (9) piloting of the final Persian version CP QOL-Child by 10 parents of children and 10 children with CP and their parents. During the translation process, the researchers were mindful that the translated content should be easily understood by parents and children with CP. The terminologies used were appropriate cross-regionally in Persian-speaking communities. 

If necessary, caregivers or the researchers were allowed to assist the child in completing the questionnaire by explaining it to them. An accessible sub-group of 20 primary caregivers was asked to complete the questionnaire twice at a 3-week interval to assess test– retest reliability. Primary caregivers were considered as those who knew the most about the child. 

Data were analyzed using SPSS (ver.16). Higher scores indicate a happier status or better well-being except for the eight items in the domain of pain and impact of disability that were originally designed in a negative direction. Principal components analysis could only be conducted on the primary caregiver-proxy data because of the small sample size of children. Correlation analyses were conducted to examine test–retest reliability, validity, and primary caregiver–child concordance on QOL scales. All analyses used a significance level of p<0.05. All scores were converted to a range from0–100. Internal consistency was estimated using Cronbach’s alpha. Item-discrimination validity was used to assess the correlation between the score of each item within the domain and other subscales. 

Confirmatory factor analysis was performed to examine if the factor structure of the translated measures were similar to the English version to assess construct validity. The association of the domain scores with other relevant information including primary caregiver age, child age, intensity of rehabilitation intervention, and GMFCS and MACS was examined by calculating the correlation coefficients. 

## Results

We followed common translation procedures and all related issues were discussed conscientiously during the translation process. For example, in the domain of social well-being and acceptance, it was debated as to whether “preschool” should be translated as “kindergarten”. Eventually, kindergarten was added to these items, as so as, the words of “happy and unhappy”, “bother”, and “ability”. In addition, the authors worked with the original developers of the questionnaire to ensure that the intended meaning was captured with the translation. Problematic items were revised based on the feedback from parents regarding the meaning and clarity of the After the revisions, none of the parents in the pilot test (20 parents of children with CP and 10 children) had further difficulty with the items. 

A total of 40 children with CP and 200 primary caregivers completed the Persian CP QOL-Child. Table I demonstrateschildren were all aged from 4–12 years and distributed evenly across GMFCS levels. Most primary caregivers had completed secondary school education, with 22% of mothers and 31% of fathers having completed a university degree. 

The mean age of these 200 children was 7.7±2.4 years. There were 103 boys (51.5%). By using the GMFCS, 22 children (11%) were classified in level I, 49 children (24.5%) in level II, 31 children (15.5%) in level III, 61(30.5%) in level IV, and 37 children (18.5%) were classified in level V. 

The children were also identified according to neuromotor classification. A total of 125 children (62.5%) with a mean age of 7.6 years were bilateral spastic CP, and 36 (18%) children with a mean age of 7.9 years were unilateral spastic CP, 10 children (5%) with a mean age of 7.6 years were ataxic, and 14 children (7%) with a mean age of 8 years were dyskinetic. There were no differences found among the CP types regarding mean age. 

Among these respondents, most of them (80%) were mothers and of the85% mothers were the main caregiver. The economic status of the family was also reported by respondent caregivers. The majority (62%) reported their family revenue and expenditure was balanced, 63 (31.5%) reported that their family revenue was insufficient. 

Most items had less than 5% missing data. However, four items in the domain of access to services, and one item in the domain of pain and impact of disability had missing values over 50% of the time.This was because primary caregivers indicated that they had never tried to access respite care and these responseswere excluded from analyses. The CP QOL-Child is designed for children across all levels of impairment and it is problematic to include items that are not appropriate for almost 50% of the sample. Seven items in different domains (3, 4, 12, 17, 41, 43, and 45) had missing values between 30–35%, due to as not having applicable values. 

Table 2 presents the mean scores and standard deviations (SD) for each domain. From the primary caregiver data, the highest domain mean score was 74.14, which was found in the domain of social well-being and acceptance. The lowest mean score (46.48) was found in the domain of pain and impact of the disability. 

Cronbach’s alpha coefficient (α) measures the average correlation among the items in the questionnaire and the number of items in the instrument. Coefficients >0.7, and probably <0.9, are recommended (25–27). For the CP QOL-Child, Cronbach’s Alpha ranges from 0.61– 0.87, which indicatesgood reliability. 

Test–retest reliability is assessed when the instrument is administered to the same population on two occasions and the results are compared by correlation. Test–retest reliability was examined at 3 weeks, excluding children who reported experiencing a significant life event since it wasfirst administered. Intraclass correlations ranged from 0.47–0.84, which indictedmoderate to good test-retest reliability. Standard Error of Measurement (SEM) was 4.8–15.27. Table 3 shows the results on test reliability. 

Validity is defined as the extent to which the instrument measures what it was intended to measure. In this study, we examined face, construct, and discriminative validity. On the primary caregiver questionnaire, the last item asked the primary caregiver how confident the child felt using a 1–9 point scale, ranging from “not at all confident” to “very confident”. A total of 160 (79.7 %) caregivers scored 6–9, revealing they had positive confidence; 18 (9%) scored 5, indicating they were neutral; and 22 (11.3%) scored 1–4, revealing negative confidence. 

The last item in the child form asked how much help was needed by the child to complete the questionnaire. A total of 27 (69.2%) children responded “no help”, 6 (15.4%) responded “a little bit of help”, 2 (5.1%) responded “quite a bit of help”, and 4 (10.3%) responded “a lot”. 

Confirmatory factor analysis could only be conducted on the primary caregiver data because of the small size of the children sample. Since a large proportion of primary caregivers did not complete the four items in the domain of access to services and one item in the domain of pain, these were not included in the factor analysis. The analysis was constrained to a seven-factor solution. Principal components analysis followed by varimax rotation was conducted on the remaining 61 items, which cumulatively accounted for 40.8% of the variance with KMO=.814 and p-value< 0.05 for the Bartlett Test. The results revealed that the factor structure of five of the seven extracted factors could be properly identified as similar to the domain structures of the original English version, including the domains of social well-being and acceptance, functioning, participation and physical health, pain and impact of disability, and access to services. Factor structure of the remaining two factors was relatively less interpretable. 

Item discrimination analysis revealed a majority of the items (80%) have high discriminating power and the Spearman correlation coefficients weresignificant except fortwo items in the pain and impact of disability (p-value<0.05). 

Construct validity requires establishing theories and testing these theories and models against the relationships of the measure. Table 4 shows the correlation between domain scores and the relevant factors. Six of the seven domains, including the domains of social well-being and acceptance, functioning, participation , physical health, emotional well-being, access to services, and family health on the primary caregiver questionnaire were moderately correlated with the GMFCS levels (r = .18–.32; p <.05), and five of the seven domains, including the domains of social well-being and acceptance, functioning, participation, physical health, family health, and emotional well-being were moderately correlated with the MACS levels (r = .13–.40; p <.05).The direction and magnitude of the correlations were consistent with the hypotheses. On primary caregiver form of questionnaire, domain scores were not significantly correlated with primary caregiver age, child age, or intensity of rehabilitation intervention for the children except for access to services and age of primary caregiver. 

## Discussion

Cerebral palsy is an umbrella term that describes a group of long-term disorders of movement and posture development that are attributed to non-progressive disturbances (2). This study attempted to translate an instrument measuring QOL for children with CP, the CP QOL-Child, into Persian. The psychometric properties of the Persian CP QOL-Child were also assessed for use in Iran. The results from this study demonstrate that the primary caregiver-proxy version of the questionnaire has high reliability and validity, and early results suggest that the child self-report version has good face validity, internal consistency, and concordance with primary caregiver-proxy version. 

The neuromotor classification, MACS and GMFCS levels revealed that the children in the study spanned across the spectrum of motor subtype and motor disability. Most of the primary caregivers who participated in the study expressed that they were confident in recording how the children felt. A majority of the participating children reported that they completed the questionnaire with no or little help. These findings are important in terms of demonstrating that the words and expressions used in the Persian CP QOL-Child are not difficult for the target populations to read and respond to. 

Regarding internal consistency, for the English version, Waters et al. (11) reported that Cronbach’s alpha ranged from 0.74–0.92 for the primary caregiver questionnaire and for the Chinese version ranged from 0.78–0.91(12), and from 0.80– 0.90 in English version, and from 0.84–0.89 for the Chinese version for child self-report questionnaires. In this study, the Persian CP QOL-Child, Cronbach’s alpha ranged from 0.61–0.87 for primary caregiver and from 0.64–0.86 for child self-report questionnaires. The above data indicate good internal consistency of the Persian version of the CP QOL-Child, comparable with that of the English version. 

With regard to test–retest reliability, for primary caregivers, ICC ranged from 0.76–0.89 for the English, 0.86–0.97 for the Chinese, and 0.47–0.84 for the Persian version (11-12). In this study, we also analyzed test– retest reliability for primary caregivers; with standard error of measurement (SEM), the SEM ranged from 4.8– 15.27 for the Persian version. In our study, a majority of items had a moderate to good discrimination index. This result indicates that items of the Persian version of CP QOL-Child have an acceptable ability to reflect differences in well-being status. 

For construct validity, the authors of the original English version assessed the relationship between the CP QOL-Child and other QOL, health, and functioning questionnaires. We took a different approach because Iran does not have a valid Child QOL questionnaire. The association of the domain scores of CP QOL-Child with other relevant information, including parentand child age, severity of motor disability, and intensity of rehabilitation intervention were analyzed. The higher the level of disability of the child and the more severe the motor deficit, the higher the reduction in the physical aspects of QOL, but independent of age of primary caregiver, age of child, and intensity of rehabilitation for the children. Schneider et al and Majnemer et al also reflected on a similarresult toours, which further showed that due to physical limitations, children with CP have physical role limitations for indoor and outdoor activities (28-29). 

A previous study explored the relationship between parental QOL and child age, and reported no significant association (30).This is confirmed by the present study as well. Moreover, this study provided additional evidence that the QOL of children with CP was not significantly correlated with the age of the primary caregiver or age of the child. These results are useful for understanding more thoroughly the impact of age on QOL. 

It is debatable whether and how higher intensity of rehabilitation is related to the promotion of motor function for children with CP. A previous study found that gross motor functions may be good predictors of the physical component of health-related QOL but poor predictors of the psychosocial component in children with CP (31).We found that for children with CP, there is no association between QOL and the intensity of rehabilitation. However, more research on the relationship between them is needed. 

Given that this instrument was primarily based on interviews with primary caregivers and children, it is encouraging that the domains are similar to other instruments, which are often based on the opinions of clinicians. Although separate principal components analysis is required to examine the structure of the child self-report questionnaire, early results suggest that the structure of the primary caregiver questionnaire and child questionnaire may be similar. 

We examined concordance in the common domains of the primary caregiver form and child self-report form of the CP QOL-Child. There was good concordance between primary caregiver proxy and child self-report data (r=0.46–0.59). Among the five comparable domains, the highest correlation found was in the domain of functioning. This finding was consistent with that observed in the original English and Chinese versions (11-12). .In contrast, the lowest correlation was in the domain of pain and impact of disability. This finding is consistent with that observed in the original English (11), but this was not the same as the Chinese version (12). These findings may reveal that thewell-being status of functioning in children is relatively easy to identify and label for primary caregivers and the children with CP. Nevertheless, there were bigger differences between the primary caregiver recognition of the extent of the child’ssocial well-being and acceptance, pain and impact of disability against those reported by the child. 

This is consistent with past QOL studies (32). Past studies have suggested that there is better agreement (>0.5) between primary caregivers and children for domains on physical health, functioning, and symptoms and poorer agreement (<0.30) for domains on social or emotional issues (32). This expected variation across domains was not seen in the current study.However, further analyses are required with a larger sample. 

Pain, which is impairment according to model of ICF, in children with cognitive impairment and CP, is a particularly relevant issue due to its high prevalence and impact on QOL (29). The literature has revealed that spasticity causes painful contractures, windswept deformity, scoliosis, and hip dislocation that result in pain and difficulty in positioning, sitting, standing, and walking (33). Similarly, in our study it was seen that children with CP have more pain and discomfort. Therefore, it is thought that especially in children with CP early physiotherapy and rehabilitation interventions can help keep pain under control and QOL can be positively affected. 

In our study as well as other studies (34), it was evident that the behavior and activities of the families in children with CP were more affected. We believe this is because social acceptance is harder in children with CP because of the physical appearance and the severity of the functional inadequacy as well as the care that is needed during the entire lifetime that greatly affects the lifestyle of the family and the relationship between family members. Activity limitations and social function restriction form a negative aspect of ICF functioning and disability are closely related with functional independence in daily living activities. 

One major limitation is that asmall number of children provided self-report data. The number of children involved in the child self-report form was limited because they were required to be between 9–12 years and have the ability to understand and respond to the questions. We are seeking to recruit more children and are investigating a short form for children with communication difficulties. Another, limitation of this study was that it was a cross-sectional study. We were unable to evaluate the ability of the Iranian CP QOL-Child in detecting changes in QOL over time. The responsiveness of the instrument will need to be examined in future studies by employing a prospective design. Moreover, the sample sizes for test–retest reliability and the child self-report data are small. 

Investigation on a larger sample of child self-report data is needed in future studies. Establishing construct validity is an ongoing process. Further research is needed to satisfy more integrated data of validity. 

In conclusion, rehabilitation goals related to increasing social function and QOL should promote and enhance health and wellbeing, rather than perpetuating the traditional emphasis on preventing and minimizing long-term disabilities and impairments in accord with the World Health Organization ICF model. Therefore, this study was necessary to develop a Persian version CP QOL-Child to identify QOL of the CP children to enable successful interventions. 

**Table 1:** Demographic Characteristics of the Studied Sample

		**Frequency**	**Percent**	
**Children** ^a^		All children (n=200)		
Age – mean (range) (years)		7.7(4-12)		
Gender	Male	103	51.5	
	Female	97	48.5	
GMFCS^b^ levels	I	22	11	
	II	49	24.5	
	III	31	15.5	
	IV	61	30.5	
	V	37	18.5	
Neuromotor Classification	Unilateral Spastic	36	18	
	Bilateral Spastic	125	62.5	
	Ataxic	10	5	
	Dyskinetic	14	7	
	Unclassified(mixed)	15	7.5	
**Primary** ** caregiver**				
Age – mean (range) (years)		34.26(18-65)		
Respondent to questionnaire	mother	170	85	
	father	4	2	
	Babysitter	7	3.5	
	Mother and father	19	9.5	
Education	Primary school	70	35	
	Secondary school	75	37.5	
	University or above	55	27.5	
Marital status	Married	191	95.5	
	Divorced/ Widow	9	4.5	
Family income and expenditure	Abundance	13	6.5	
	Balance	124	62	
	Insufficiency	63	31.5	

**Table 2:** Descriptive Statistics of the CP QOL-Child Domain Scores on a 9-Point Scale

**Domain of CP QOL-Child** **Primary caregiver (n= 200)**	**Mean** **(scores converted to 0–100** **)**	**Standard Deviation**	**Domain of Range**
**Social well-beingand acceptance**	74/14	14/34	71/59
**Functioning**	63/30	14/46	82/29
**Participation and physical health**	66/25	15/80	87/50
**Emotional well-being**	70/50	16/12	79/17
**Access to services**	56/06	21/30	95
**Pain and impact of disability**	46/48	20/69	85/71
**Family health**	48/61	27/55	100

**Table 3:** Results of Internal Consistency, Test–Retest Reliability, Item-Discrimination, and Primary Caregiver–Child Concordance of the CP QOL-Child

**Domain of CP QOL-Child**	**Cronbach’s alpha (Parent report)** ** (n= 200)**	**Cronbach’s alpha (Child report) ** **(n ** **= ** **40)**	**ICC (95% CI) (Parent report) 3wk (n ** **=** **20)**	**Standard Error of Measurement (SEM)**	**item-discrimination** ** index (Parentreport) (n ** **=** **200)**	**Correlation between parent and child reports (n ** **= ** **40)**
**Social well-being and acceptance (12 items)**	0.87	0.75	0.84(0.59-0.94)	5.16	0.39-0.73	0.29
**Functioning (12 items)**	0.82	0.68	0.73(0.33-0.89)	6.75	0.23-0.63	0.59^a^
**Participation and physical health** **(11 items)**	0.85	0.86	0.79(0.46-0.92)	4.80	0.35-0.58	0.56^a^
**Emotional wellbeing (6 items)**	0.81	0.73	0.47(-0.34-0.79)	10.30	0.28-0.71	0.46^a^
**Access to services (12 items)**	0.78	b	0.57(-0.09-0.83)	11.97	0.21-0.68	b
**Pain and impact of disability (8 items)**	0.61	0.64	0.74(0.35-0.9)	10.29	0.11-0.53^c^	0.13
**Family health (4 items)**	0.85	b	0.71(0.28-0.89)	15.27	0.65-0.71	b

ICC, intraclass correlation coefficient; CI, confidence internal

a p<01; bindicates domains that are not included in child self-report version; c except for two items.

**Table 4:** Correlations between Domains of Primary Caregiver Questionnaire of CP QOL-Child and Relevant Factors

	**Age of primary** **Caregiver**		**Age of child**		**Intensity of** **Rehabilitation**		**GMFCS Levels**		**MACS** **Levels**	
**Domain of CP QOL-Child**	Correlation Coefficient	p-value	Correlation Coefficient	p-value	Correlation Coefficient	p-value	Correlation Coefficient	p-value	Correlation Coefficient	p-value
**Social well-being and acceptance**	.023	.751	-.030	.674	-.098	.182	-.21	<.05	-.24	<.05
**Functioning**	-.033	.645	-.023	.741	-.039	.593	-.32	<.05	-.40	<.05
**Participation and physical health**	-.032	.655	-.018	.795	-.101	.166	-.27	<.05	-.24	<.05
**Emotional well-being**	-.117	.103	-.108	.127	-.083	.259	-.23	<.05	-.27	<.05
**Access to services**	-.150	<.05	.012	.870	.105	.152	-.18	<.05	-.09	-
**Pain and impact of disability**	.000	.992	.044	.532	.019	.794	.07	-	-.03	-
**Family health**	-.053	.456	.105	.140	.119	.106	-.2	<.05	-.13	<.05
